# A Nexus Model of Restricted Interests in Autism Spectrum Disorder

**DOI:** 10.3389/fnhum.2020.00212

**Published:** 2020-06-03

**Authors:** R. McKell Carter, Heejung Jung, Judy Reaven, Audrey Blakeley-Smith, Gabriel S. Dichter

**Affiliations:** ^1^Institute of Cognitive Science, University of Colorado Boulder, Boulder, CO, United States; ^2^Department of Psychology and Neuroscience, University of Colorado Boulder, Boulder, CO, United States; ^3^JFK Partners, Department of Psychiatry and Pediatrics, University of Colorado Anschutz Medical Campus, Aurora, CO, United States; ^4^School of Medicine, Carolina Institute for Developmental Disabilities, The University of North Carolina at Chapel Hill, Chapel Hill, NC, United States; ^5^Department of Psychiatry, The University of North Carolina at Chapel Hill, Chapel Hill, NC, United States; ^6^Department of Psychology and Neuroscience, The University of North Carolina at Chapel Hill, Chapel Hill, NC, United States

**Keywords:** autism, restricted interests, cognitive neural development, fMRI, social perception

## Abstract

Restricted interests (RIs) in autism spectrum disorder (ASD) are clinically impairing interests of unusual focus or intensity. They are a subtype of restricted and repetitive behaviors which are one of two diagnostic criteria for the disorder. Despite the near ubiquity of RIs in ASD, the neural basis for their development is not well understood. However, recent cognitive neuroscience findings from nonclinical samples and from individuals with ASD shed light on neural mechanisms that may explain the emergence of RIs. We propose the nexus model of RIs in ASD, a novel conceptualization of this symptom domain that suggests that RIs may reflect a co-opting of brain systems that typically serve to integrate complex attention, memory, semantic, and social communication functions during development. The nexus model of RIs hypothesizes that when social communicative development is compromised, brain functions typically located within the lateral surface of cortex may expand into social processing brain systems and alter cortical representations of various cognitive functions during development. These changes, in turn, promote the development of RIs as an alternative process mediated by these brain networks. The nexus model of RIs makes testable predictions about reciprocal relations between the impaired development of social communication and the emergence of RIs in ASD and suggests novel avenues for treatment development.

## Background

Restricted interests (RIs) in autism spectrum disorder (ASD) are clinically impairing interests of unusual focus or intensity that are a subtype of the restrictive and repetitive behaviors symptom domain of ASD (American Psychiatric Association, 2013). RIs are strongly associated with ASD (Gal, 2011), are typically challenging to treat (Dawson et al., 2010), and are described in some of the earliest accounts of ASD (Kanner, 1943; Asperger, 1944). Additionally, they are less often studied than social communication symptoms of ASD (Richler et al., 2007), making them a promising avenue for improving our understanding of ASD. The manifestations of RIs are highly variable in expression and intensity (Baron-Cohen and Wheelwright, 1999), and early descriptions of RIs in ASD characterized their content or topic area (Kanner, 1943; Asperger, 1944; Baron-Cohen and Wheelwright, 1999; South et al., 2005), though the focus of RIs often change over time and are not easily semantically categorized (Klin et al., 2007). Although the neural bases of RIs are not well understood, recent research has highlighted that RIs engage mesolimbic motivational brain systems (Clements et al., 2018), compete with social stimuli for attentional resources (Sasson and Touchstone, 2014), and prompt effort expenditure to seek out RIs (Traynor et al., 2019) in individuals with ASD.

The goal of this paper is to provide a framework to explain the neural mechanisms underlying the development of RIs. We propose a nexus model of RIs as a brain-based developmental bridge between social communication impairments and RIs in ASD. The nexus model emphasizes that RIs may be conceptualized as a preferred mode of engaging with the world (Baron-Cohen et al., 2003; Klin et al., 2007) that first emerges early in life and suggests that RIs may reflect cognitive abilities and supporting neural systems that are strengthened during development relative to those of individuals without ASD. RIs involve altered development of both the social cognition and reward systems; they are linked and develop in concert. Without social motivation, the perceptual and modeling areas do not develop because social stimuli do not elicit behavior to attend or approach. Without social cognition, social motivation cannot develop optimally because actors and predictions must be neurally represented to be motivating. We recognize the extensive literature documenting associations between the reward system and RIs [e.g., (Dichter et al., 2012; Cascio et al., 2014; Kohls et al., 2018; Harrop et al., 2019) and the role motivation plays in the formation of RIs (Chevallier et al., 2012; Dichter, 2018)]. The mechanism we propose here reflects a complementary framework addressing the influence of motivation on RI development (Dawson et al., 2005; Clements et al., 2018; Dichter, 2018): namely that RIs arise as motivation and cognitive systems are mutually constraining during development in ASD. We hold the description of the interaction between reward systems and social cognitive development for later work. Here, we focus on how the altered development of social cognitive systems could produce RIs.

### The Nexus Model of Social Cognition Highlights the Role of the Temporal-Parietal Junction in Social Processing

**FIGURE 1:**
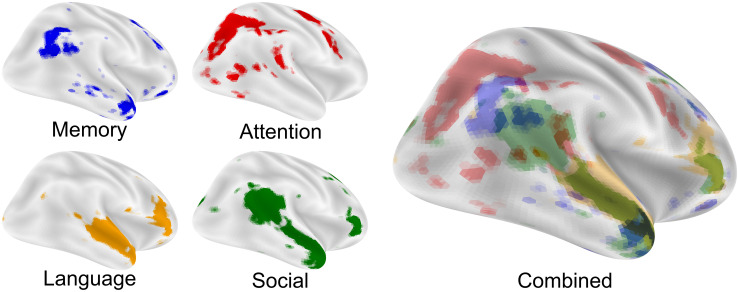
The nexus model of social function. The nexus model hypothesizes that complex social functions arise where memory, attention, language, and social processing come into close proximity and can be combined. Each image is an FDR corrected reverse-inference (likelihood of term given activation) statistical image from neurosynth.org overlaid on an inflated cortical surface using nilearn. Maps were taken from the 200-topic cognitive maps: Memory is topic 28; Attention is topic 64; Language is topic 93 and Social is topic 145. All maps were downloaded in the summer of 2019 from the July 2018 LDA 200-topic model from neurosynth.org based on 14,371 studies.

The nexus model of social cognition (Carter and Huettel, 2013) describes the convergence of cognitive processing streams that are related to preferred modes of engagement. As an example, the temporal-parietal junction (TPJ) is a multimodal brain region involved in attention (Mitchell, 2008; Corbetta and Shulman, 2011; Geng and Vossel, 2013), memory (Berryhill et al., 2007; Cabeza et al., 2012), language (Binder et al., 2009; Donaldson et al., 2015; Redcay et al., 2016), and social processing (Gallagher and Frith, 2003; Saxe and Kanwisher, 2003; Young et al., 2010) functions (Figure 1). This model asserts that novel, complex, predictive social cognitive functions emerge from the functional and anatomical intersection of attention, memory, language, and social processing streams in the brain (Carter and Huettel, 2013), and the combination of output from these processes provides a means to predict future social action by others (Hill et al., 2017). Each constituent process has its own developmental progression that must be successfully completed before it can be combined with other necessary functions to support complex social cognition and social communication (Carter and Huettel, 2013). Given this, a change in the developmental progression of these processing streams may give rise to alternative preferred modes of engagement.

**FIGURE 2:**
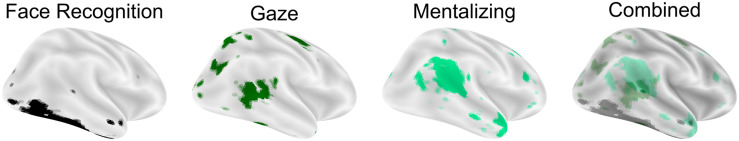
Hierarchical construction of flexible social cognition. Social cognitive processes begin with face recognition on the lateral occipital surface and more superior/anterior regions representing more complex aspects of social cognition. Images are surface overlay of a reverse-inference maps for the terms “face recognition,” “gaze,” and “mentalizing” downloaded from neurosynth.org in the summer of 2019 (based on 14,371 studies).

The development of complex social processes necessarily depends on the prior development of simpler social processes. These social building blocks are bootstrapped through interactive specialization (Johnson, 2011), by which early experiences produce responses that drive the development of more complex functions. For example, analogous to social development, regions of the brain anatomically close to sensory areas drive the functional specialization of regions further from sensory areas as development progresses. Representations grow increasingly abstract as visual information travels from V1 along the ventral visual stream, supporting object recognition (Goodale and Milner, 1992; DiCarlo et al., 2012). A similar increase in complexity is evident for social cognition. Meta-analyses of neuroimaging data show a pattern of activation in response to complex visual social stimuli that predict future actions (Carter and Huettel, 2013). Moving superior and anterior from the lateral-occipital face processing area, cortical regions are sensitive to more complex and abstracted social-cognitive representations (Figure 2). This pattern of increasing complexity in representations of social stimuli is not thought to exist at birth but occurs sequentially in a developmental cascade (Johnson, 2011). In this way, development proceeds sequentially, focusing at first on simpler processes which form the basis for more complex behaviors (Nelson, 1999). This progression is constructivist in that the development of each cognitive function depends upon previous and less complex and abstract functions (Quartz and Sejnowski, 1997), but also relies on nativist principles. In particular, patches of cortex involved in social processing are reliably associated with specific computations (Srihasam et al., 2014; Livingstone et al., 2017) and are consistently anatomically located across individuals. Functions within these same areas are also subject to change by experience (Dehaene et al., 2010) in a manner that relies upon their particular functional network (Mahon and Caramazza, 2011), commonly referred to as experience-dependent plasticity. Social development thus relies on the emergence of specific brain functions at birth and on the reinforcing interactions that occur as these functions are successfully used. The neural development of complex social cognition recapitulates the order of social-developmental milestones in infancy and childhood with implications for ASD (Shultz et al., 2018): should the development of each social area of cortex depend on earlier, simpler areas, as is predicted by interactive specialization, decreased orienting to social stimuli, or any deficiency in the ability to represent those stimuli, may leave areas of cortex critical for social cognition underdeveloped.

### Altered Developmental Trajectories Lead to Preferred Modes of Engagement in ASD

**FIGURE 3:**
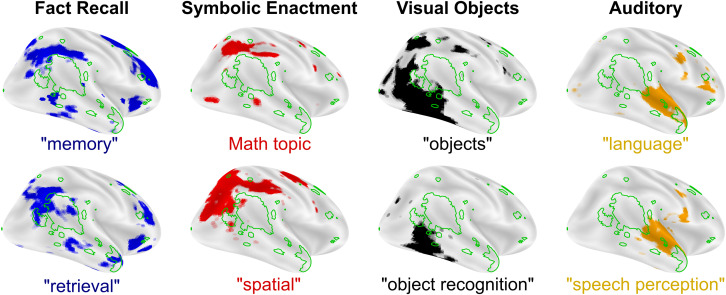
Cognitive processes and preferred modes of engagement. Undeveloped complex social functions like mentalizing (Neurosynth reverse inference “mentalizing,” green outline) may leave areas of cortex open to expansion from neighboring cognitive functions that can be mapped to preferred modes-of-engagement for RIs in autism. FDR corrected, reverse inference (likelihood of term given activation) statistical images from neurosynth.org are overlaid on inflated cortical maps using Nilearn for each term in quotes. “Math topic” is Topic number 2 from version 5 (July 2018) of the Neurosynth set of LDA derived topic maps with prominent terms like: problem(s), arithmetic, solving, and calculation. All maps were downloaded in the summer of 2019 (based on 14,371 studies).

In the absence of valued (Chevallier et al., 2012; Dichter et al., 2012; Abrams et al., 2013; Chen et al., 2015) or easily interpretable (Sasson et al., 2013; Hohwy and Palmer, 2014) social input, neural systems supporting complex social cognitive hierarchies fail to develop, leaving cortical areas that are critical for social cognitive functions potentially capable of assuming, at least in part, alternative functions. When typical input to a brain region is disrupted, the region often takes on meaningful functions using alternative or indirect input. For example, neurons in visual cortex in the congenitally or early blind are selectively active during braille reading (Sadato et al., 1996, 2004; Cheung et al., 2009), speech processing (Kujala et al., 2005; Hertrich et al., 2009; Dietrich et al., 2013), or even patterned electrical stimulation to the tongue (Ptito et al., 2005; Matteau et al., 2010). For reasons that are not clearly understood, social stimuli activate reward systems, including the dorsal and ventral striatum and medial prefrontal cortex, to a lesser degree in ASD (Clements et al., 2018; Kohls et al., 2018; Supekar et al., 2018). A critical implication of the nexus model of RIs in ASD is that when rewarding responses to social stimuli are dampened early in development, areas of the brain that govern more complex social cognitive processes will develop in a delayed fashion. Critically, experience-dependent and experience-expectant social-cognitive brain areas would show altered developmental trajectories and thus would be relatively more responsive to other, non-social sources of input (Butz et al., 2009; Holtmaat and Svoboda, 2009). In the same way that visual cortex utilizes new inputs for spatial processing when visual information is unavailable, areas of cortex that typically predict future states of complex social stimuli may instead make predictions about RI-related stimuli. Extending the nexus model, any of the cognitive processes that typically neighbor social processing in the cortex (Figure 3) may expand into underdeveloped social areas of the brain. This biased flexibility in processing is inherent in the theory of interactive specialization and may provide a mechanism for better characterizing regional brain functions. The interactive development of cortex is also hypothesized to underlie the development of language-specific cortical areas. Through a mechanism described as cortical recycling, Dehaene and colleagues hypothesize that the visual word form area develops from cortical tissues previously devoted to face and object processing (Dehaene and Cohen, 2011). This link between face and language processing is akin to the repurposing of social communication brain regions for RI processing.

The nexus model of RIs suggests that brain regions that typically mediate social communication, including most centrally the TPJ, demonstrate a functional shift during development towards processing information related to RIs. This co-opting highlights a perspective that RIs reflect a preferred mode of engaging with the world in individuals with ASD. Whereas early descriptions of RIs in ASD characterized the specific content or topic area of RIs (Kanner, 1943; Asperger, 1944; Baron-Cohen and Wheelwright, 1999; South et al., 2005) (e.g., folk physics, objects, or machines), a broader perspective on RIs (as a preferred mode of engagement) accounts for changes in RIs over time. It also accounts for the fact that RIs are not always easily categorized semantically, and emphasizes types of interactions that may be part of an individual’s RI (e.g., fact memorizing, counting, spatial manipulation, and categorizing), (Attwood, 2003; Baron-Cohen et al., 2003; South et al., 2005; Klin et al., 2007). The perspective that RIs reflect a preferred mode of engagement suggests that RIs reflect cognitive abilities that are relatively enhanced and consequently pursued (Spiker et al., 2012) and represents a more parsimonious account of RIs as reflecting altered functioning of brain systems that mediate higher-order cognitive processes in individuals with ASD, as will be described in the next section.

### A Nexus Model of RIs in ASD

In this section, we focus on four cognitive functions represented in cortical and neighboring social processing brain regions that may be linked to preferred modes of engagement and RIs in ASD: (1) memory (e.g., fact recall RIs), (2) spatial attention (e.g., calculating, building, and mapping RIs), (3) object processing (e.g., object expertise and reading RIs), and (4) auditory processing (e.g., tone and speech RIs).

Memory recall is associated with activation in the posterior parietal cortex, an area of the brain that anatomically neighbors mentalizing functions in the TPJ (Figure 3, left). There are some documented changes in the hippocampus in ASD (Barnea-Goraly et al., 2014; Trontel et al., 2015; Cooper et al., 2017). However, if the hippocampus were the primary driver of memory deficits in ASD, these deficits found in individuals with ASD would be expected to be general in nature. Instead, the majority of studies that find changes in memory in ASD report specific impairments, and not a general deficiency (Boucher, 1981; Renner et al., 2000; Toichi and Kamio, 2002, 2003). Higher functioning individuals with ASD perform well on standard cued recall and paired-association learning (Boucher and Warrington, 1976; Minshew and Goldstein, 2001; Williams et al., 2006) and recall tasks involving non-social items, such as buildings and leaves, but have been found to perform poorly on face memory tasks (Blair et al., 2002). Neurobiologically, specific memory deficits like these are unlikely to be due to changes in the hippocampus, as patients with hippocampal lesions show broad memory deficiencies (Winocur and Weiskrantz, 1976; Shimamura and Squire, 1988; Holdstock et al., 2002). Instead, these specific deficits are better attributed to memory recall functions in the posterior parietal cortex (Hadjikhani et al., 2004), which, when lesioned, produce subtle and selective memory deficits resembling episodic memory impairments (Boucher and Mayes, 2012). Thus, parietal cortical mechanisms are implicated in memory-based RIs and improved memory recall observed in ASD. Developmentally, early changes in motivation or exposure to social stimuli may alter development of the lateral surface of the cortex in ASD. Variations in the precise timing of these changes would result in memory alterations in ASD, ranging from memory deficiencies to memory enhancement (Rimland, 1978), which may result in memory-related RIs in some individuals with ASD.

Second, spatial attention, numeracy, and timing are related to automatic and volitional attentional control (Hartje, 1987; Walsh, 2003), and these functions each produce activity adjacent to social processing brain areas in the intraparietal sulcus (Hubbard et al., 2005; Ansari et al., 2007; DeWind et al., 2015) (Figure 3, left-middle) that are themselves implicated in ASD (Allman et al., 2011b; Dichter, 2012). Some individuals with ASD exhibit evidence of compromised functioning of attentional streams reflected in less frequent (Baranek, 1999) and slower (Wainwright and Bryson, 1996; Keehn et al., 2010) orienting. Others studies, however, have documented evidence of enhanced spatial attention in ASD (Mottron et al., 2006), reflected in faster responses on a conjunctive visual search task (Jarrold et al., 2005) and greater accuracy in the embedded figures (Jolliffe and Baron-Cohen, 1997) and block design (Tymchuk et al., 1977; Siegel et al., 1996) tasks. There are also examples of compromised numeracy (Meaux et al., 2014) and timing (Allman et al., 2011a) in ASD. In functional neuroimaging studies of selective attention, there is evidence that activation in the dorsal attention stream (including the intraparietal sulcus) is greater or more variable in ASD (Belmonte and Yurgelun-Todd, 2003). A popular theory for explaining better visual performance in some cases is that individuals with ASD have enhanced perceptual function (Mottron et al., 2006), focusing on simpler, rather than more abstract, dimensions (Bertone et al., 2005) or on local, rather than global, configurations (Rinehart et al., 2000) of visual stimuli. The nexus model of RIs would hypothesize that these differences may reflect improved spatial attention, numeracy, and timing due to expanded representations into cortical brain regions, including specifically the TPJ.

Third, object sensitive areas of the brain are arranged along the lateral occipital surface of the brain (Figure 3), adjacent to areas of the brain that respond to faces, emotions, and eye-gaze (Figure 2). Enhanced visual perception has been reported in a number of contexts in ASD, including fine pattern discrimination (Plaisted et al., 1998a), conjunction search (Plaisted et al., 1998b), orientation (Bertone et al., 2005), and other tasks (Dakin and Frith, 2005). Performance improvements in visual perception in ASD tend to be constrained to lower-order abilities, whereas deficits in integrative and holistic tasks are commonly reported in ASD (Happé and Frith, 2006). Brain areas that mediate many of these enhanced processes border the lateral occipital face area of the brain, raising the possibility that small differences in performance on these tasks in early childhood may developmentally expand these cortical representations, leading to expanded lower-order visual processing and by extension reduced focus on social stimuli. In fact, a common occurrence in ASD is hyperlexia, a syndrome that relies on character and word recognition areas of the brain in the ventral visual pathway that borders face processing brain areas (Ostrolenk et al., 2017), offering a potential mechanistic explanation for the co-occurrence of diminished social function and hyperlexia in ASD.

Relatedly, an early hypothesis for social communication deficits in ASD is a diminished ability to process faces (Dawson et al., 2002). Although this deficit is not universally true, individuals with ASD tend to have a diminished ability to recognize unfamiliar faces (Weigelt et al., 2012; Tang et al., 2015), which in turn may also impact social functions including social memory (Ewbank et al., 2017), social eyescan paths (Pelphrey et al., 2002), social emotion recognition (Adolphs et al., 2001; Bal et al., 2010), and face inversion effects (Scherf et al., 2008), though there may be compensation for familiar or enhanced stimuli (Pierce et al., 2004; Pierce and Redcay, 2008). Proposed explanations for diminished face recognition in ASD are that it reflects lower-level perceptual impairments (Behrmann et al., 2006), reduced generalization (Plaisted, 2001), and enhanced perceptual function (Mottron et al., 2006) [for review see Mottron et al. (2009); Thye et al. (2018)]. Although low-level perceptual changes explain enhanced functions in ASD, understanding higher-order impairments has traditionally relied on an additional proposed mechanism, such as diminished veridical mapping and weak central coherence. While not typically described in terms of a specific brain mechanism, these compromised higher-order processes are mediated by the TPJ (Dakin and Frith, 2005). As in memory and attentional RIs, this heterogeneity of visual phenotypes in ASD hints at variations in developmental trajectories that depend on preferred modes of engagement and the development of lower-level processes that support a specific RI class. Because object and text sensitive brain areas (Figure 3, right-middle) abutt brain regions specialized for face and emotional processing, they are positioned to expand in cases where face processing is compromised during development. This link between objects/text and social brain areas predicts that face processing areas of the brain may respond preferentially to RIs that focus on text and objects. For example, a case study of a boy with ASD found greater brain activation to his RI (a Digimon^TM^ cartoon character) in the fusiform face area (FFA) compared to human face stimuli (Grelotti et al., 2005). This study demonstrated the possibility of plasticity in FFA responses in ASD and served as evidence that lower-level face processing brain regions may develop to respond to non-face stimuli in ASD. Foss-Feig et al. (2016) compared responses to object RIs of individuals with ASD to responses to intense interests of typically developing individuals. Both groups exhibited greater activation in the FFA in response to their own RIs. In addition, activation was more robust for object RIs in ASD compared to responses of typically developing individuals. Numerous additional studies have shown that the FFA of individuals with ASD responded more to non-social stimuli (Pierce and Redcay, 2008; Perlman et al., 2011; Foss-Feig et al., 2016; Whyte et al., 2016). We include cartoon and video-game characters as object RIs since their treatment is mechanistic in nature. In typically developing individuals, the study of expertise has shown increased activation in the FFA when viewing the focus of their expertise (Gauthier et al., 2000), supporting the argument for repurposing based on interest more broadly. We hypothesize that this modified development of functional specialization can drive changes in the ascending social cognitive hierarchy, reshaping dynamic social cognitive areas of the brain to respond to dynamic aspects of RIs.

Fourth, auditory areas of the brain are organized along the superior temporal sulcus just anterior to mentalizing areas in the TPJ (Figure 3, right). Auditory processing in ASD is atypical in a number of ways, including hyper- and hypo-sensitivity and unusual abilities like absolute pitch [for review see Samson et al. (2006)]. As with visual perception, individuals with ASD commonly show enhanced lower-order perception (Bonnel et al., 2003) and diminished abilities when working with complex voice stimuli (Gervais et al., 2004). It is, however, important to note that, as with visual stimuli, familiar voice recognition is not impaired in ASD (Boucher et al., 2000). Impairments in processing of complex auditory stimuli in ASD include both spectral and temporal aspects with compensation occuring when lower-order auditory processing may be leveraged to complete the task (Samson et al., 2006). Accordingly, auditory RIs in ASD are often related to music (Sacks, 2008), for which preferences are similar to typically developing individuals (Boso et al., 2009). In fact, impaired functional brain connectivity during speech processing may be improved by a transition to singing in ASD (Sharda et al., 2015), consistent with greater emotional comprehension for music that has been observed in ASD (Molnar-Szakacs and Heaton, 2012). In line with these findings, neuroimaging studies show a familiar pattern: areas of the brain that typically respond to speech respond more strongly to music in ASD (Lai et al., 2012). This cortical shift from processing social stimuli to processing non-social stimuli parallels the findings in the ventral visual stream discussed above, and may reflect a co-opting of typically social processing pathways in favor of responding to RIs. This repurposing of neural regions again indicates that areas of the brain that typically respond to voices would respond preferentially to auditory RIs in ASD.

Although we have described each of these cognitive functions as separate, they likely compete for processing resources during development, suggesting that both in ASD and typically developing individuals these processes may be more or less dominant. It also suggests that combinations of preferred modes of engagement would be common, as long as preferred functions related to RIs are mediated by brain areas typically used by functions that have been diminished. For example, as noted in this section, responses to music may be mediated by brain regions that typically respond to voices and responses to text or objects may be mediated by brain regions that typically respond to social stimuli such as faces. The increased processing of non-social stimuli in lower-order social processing brain areas would drive the development of non-social functional specialization in integrative brain areas, including the TPJ. We next explore preliminary evidence for such an outcome.

### Restricted Interests as Both Strengths and Challenges

Restricted interests (RIs) are a prominent characteristic of ASD that, by definition, cause impairment. RIs interfere with social development (Attwood, 2003; Turner-Brown et al., 2011), restrict the experiences of young children with ASD (Pierce and Courchesne, 2001) and interfere with learning adaptive behaviors (Koegel and Covert, 1972; Koegel et al., 1974; Varni et al., 1979). Direct tradeoffs between engaging with social stimuli and RIs have been observed in eye-tracking experiments (Sasson et al., 2008, 2011; Sasson and Touchstone, 2014; Unruh et al., 2016) and in reports of peer engagement (Boyd et al., 2007; Jordan and Caldwell-Harris, 2012) in individuals with ASD. RIs and social stimuli may directly compete for neural and attentional resources (Richey et al., 2014; Unruh et al., 2016). However, RIs may also represent areas of cognitive strengths (Grigorenko and Sternberg, 1997; Céline et al., 2000; Armstrong, 2011, 2014; Caldwell-Harris and Jordan, 2014). If social processing areas of the brain develop to preferentially respond to a preferred mode of engagement, then performance within that mode may exceed performance in other modes and may exceed that of typically developing individuals. Savant syndrome is a condition in which individuals who show substantial performance deficits in many areas have very specific areas in which they excel. This condition occurs in only ∼10% of individuals with ASD (Rimland, 1978) but approximately half of individuals with savant syndrome also have ASD (Treffert, 2009). Rimland’s 1978 survey found prodigious memory in nearly all individuals with savant syndrome with 53% focused on music, 40% on memorization, 25% on mathematical or calculating skills and 19% on art, categories that fit well with the nexus model of RIs. Even in individuals with ASD without savant syndrome, there are performance increases in narrow skill areas (Tymchuk et al., 1977). Approximately half of individuals with ASD show substantially better performance on the block design portion of intelligence tests compared to 2% of typically developing individuals (Caron et al., 2006). Enhanced performance on the block design task for individuals with ASD is generally attributed to enhanced perceptual function (Caron et al., 2006), which is also consistent with enhanced processing of auditory stimuli such as pitch and height discrimination of pure tones (O’Riordan and Passetti, 2006), complex tones (Heaton et al., 2008) and musical material (Mottron et al., 2000). Enhanced block-design performance may be due to additional processing resources in the spatial attention areas of the brain. Accordingly, individuals with non-spatial/symbolic preferred modes of engagement may show performance peaks on other tasks matched to their preferred mode of engagement.

Temple Grandin describes the importance of helping a child with ASD to find their strengths (Grandin, 2011), and indeed RIs can be leveraged to improve social functioning in ASD intervention contexts (Kasari et al., 2006; Boyd et al., 2007; Schertz and Odom, 2007). RIs have also been shown to be positive targets for therapy (Charlop-Christy and Haymes, 1996; Carnett et al., 2014; Patten Koenig and Hough Williams, 2017) and can positively affect social abilities when they are incorporated into treatment (Boyd et al., 2007; Koegel et al., 2012, 2013; Harrop et al., 2019). The Early Start Denver model, for example, is an approach that aims to develop skills by rewarding pro-social behaviors during early developmental periods when repurposing of neural pathways is most likely (Dawson et al., 2010). In the film *Life Animated*, a child with ASD has an RI that is focused on sidekicks in Disney movies. His parents were able to engage with their son by using the voices of these characters and through this interaction, gradually encouraged him to increase his social interactions more broadly. Modern virtual reality approaches raise a new possibility that dynamic and interactive worlds can be produced based on individual RIs (Thies et al., 2016), potentially enabling the automated development of teachers based on preferred mode of engagements that could dramatically improve the lives of some individuals with ASD.

### Challenges to the Nexus Model of RIs, Future Directions, and Conclusions

There are several clear challenges to the nexus model of RIs. First, there is mixed support for a relationship between RI intensity and social impairment. While some studies of RIs have found such a relationship (Turner-Brown et al., 2011; Jordan and Caldwell-Harris, 2012), others have failed to do so (Lam et al., 2008). Whereas the relationship between social and non-social behaviors may be complex and non-linear, neuroimaging studies testing the nexus model of RIs would need to be sufficiently powered to address this potential inconsistency. Second, clearly brain regions outside of social processing systems are implicated in ASD. For example, recent work in a large, multi-study sample did not find reduced activation in the TPJ during a false belief task in individuals with ASD (Dufour et al., 2013). Third, a preference for systematizing has been shown to be greater in individuals with ASD (Turner-Brown et al., 2011), but it is not clear how this preference relates to the nexus model. Finally, rather than a co-opting of social communication brain networks, RIs have also been hypothesized to reflect impaired executive function that results in perseverative behaviors (Russell, 1997; Turner, 1999), an account that differs in testable ways from the implications of the nexus model of RI development. A related hypothesis suggests that insistence on sameness and habitual behavior could be due to changes in the basal ganglia (Calderoni et al., 2014; Sinha et al., 2014; Kohls et al., 2018), which is related to habitual behaviors in typically developing individuals.

A hypothesized framework whereby RIs reflect canalization of social processing brain systems during development in ASD makes a number of testable predictions. First, RIs should reflect preferred modes of engagement rather than specific topics. While there is evidence for this conceptualization of RIs (Baron-Cohen et al., 2003; Klin et al., 2007), future RI studies should assess modes of engagement to allow for a formal comparison of this and more topic-driven models. Second, individuals with a particular RI should have thicker or expanded cortical gray matter in brain areas associated with processing that specific RI. For example, an individual with a symbolic enactment RI (e.g., counting) should show greater gray-matter thickening or larger cortical areas near the intraparietal sulcus. Additionally, areas of the brain typically responsive to social stimuli (especially those that are spatially proximal) should respond to RIs to a greater degree in individuals with ASD. For example, the TPJ would be hypothesized to show more activation to spatial attention and numeric tasks than in typically developing individuals given that their preferred mode of engagement is attention-oriented. Lastly, using brain network analysis, the nexus model of RIs would predict that the processing stream associated with the preferred mode of engagement should be more locally integrated than processing streams associated with less preferred modes of engagement. Similar functional processing streams (Margulies et al., 2016) as well as their alteration in ASD (Hong et al., 2019) lend early support for this possibility.

## Conclusion

We have described a possible developmental neural mechanism that may lead to RIs in which the altered development of social communication processing brain areas result in preferred modes of engagement and ultimately RIs. The nexus model of RIs suggests testable hypotheses about the reciprocal relation of RIs and impaired social communication and highlights novel avenues for treatment development. Most critically, although ASD research has often addressed social communication impairments and restricted and repetitive behaviors as distinct symptom domains, the nexus model of RIs suggests that social communication skills and RIs may be functionally linked during development, and that any comprehensive intensive early ASD intervention must address both social communication and RIs to be maximally effective.

## Data Availability Statement

Meta analyses figures were generated from publicly available utilities (neurosynth.org).

## Author Contributions

All authors participated in the preparation and review of the manuscript. RC generated figures.

## Conflict of Interest

The authors declare that the research was conducted in the absence of any commercial or financial relationships that could be construed as a potential conflict of interest.

## Abbreviations

ASD: autism spectrum disorder
FFA: fusiform face area
RI: Restricted Interest
TPJ: Temporal-Parietal Junction.
